# Biocompatibility of an experimental endodontic sealer (Resil) in comparison with AH26 and AH-Plus in rats: An animal study

**DOI:** 10.34172/joddd.2022.019

**Published:** 2022-10-15

**Authors:** Hengameh Ashraf, Parviz Shafagh, Fatemeh Mashhadi Abbas, Soolmaz Heidari, Hossein Shahoon, Amin Zandian, Leila Aghajanpour, Saeede Zadsirjan

**Affiliations:** ^1^Department of Endodontics, School of Dentistry, Shahid Beheshti University of Medical Sciences, Tehran, Iran; ^2^Endodontist, Private Practice, Tehran, Iran; ^3^Department of Pathology, School of Dentistry, Shahid Beheshti University of Medical Sciences, Tehran, Iran; ^4^Department of Operative Dentistry, Dental Caries Prevention Research Center, Qazvin University of Medical Sciences, Qazvin, Iran; ^5^Department of Oral and Maxillofacial Surgery, Faculty of Dentistry, Shahed University, Tehran, Iran; ^6^Dental Research Center, Restorative Department, School of Dentistry, Shahid Beheshti University of Medical Sciences, Tehran, Iran; ^7^Stem Cell Preparation Unit, Farabi Eye Hospital, Teheran University of Medical Sciences, Teheran, Iran

**Keywords:** Biocompatibility, Endodontics, Epoxy resin-based root canal sealer, In vivo studies

## Abstract

**Background.** This experimental study sought to assess the biocompatibility of Resil, an experimental epoxy resin-based sealer, in comparison with AH26 and AH-Plus sealers in rats.

**Methods.** Twelve male Wistar rats weighing 400 to 500 grams were evaluated in this experimental study. Four polyethylene tubes containing Resil, AH-Plus, AH26 sealers, and an empty tube were implanted subcutaneously in rats. The degree of inflammation, type of inflammatory cells present, foreign body reaction, quality of connective tissue, and presence of fibrotic capsule were evaluated histopathologically at 7 and 30 days after implanting the tubes to assess the biocompatibility of sealers. Data were analyzed using the Chi-square test.

**Results.** At 7 days, the degree of inflammation in Resil group was almost similar to AH26 group, and 66.7% of rats showed moderate inflammation. AH-Plus group showed less inflammation than Resil and AH26 (50% of rats showed low degree of inflammation), At 30 days, the inflammatory status of all groups was the same, and 83.3% of rats showed very low degree of inflammation. The inflammatory response during the experiment decreased from day 7 to day 30 in all groups. The neutrophil count (*P*=0.00), fibrotic capsule (*P*=0.01) and the amount of granulation tissue (*P*=0.05) significantly decreased from day 7 to day 30 in Resil group.

**Conclusion.** Resil sealer showed appropriate biocompatibility at 7 and 30 days after subcutaneous implantation in rats, comparable to AH26 and AH-Plus. Clinical studies are required to confirm these results.

## Introduction

 Pulp involvement necessitates endodontic treatment, which encompasses three phases of diagnosis and treatment planning, cleaning and shaping of the root canal system, and root canal filling. Complete sealing of the root canal system with root filling materials is a fundamental step in root canal treatment. Gutta-percha in combination with endodontic sealer is extensively used for root canal obturation. Application of sealer is imperative for higher adaptation of gutta-percha with root canal walls, sealing of dentinal tubules, and benefitting from its antimicrobial properties. For ideal root canal filling, the canal must be sealed at both the coronal and apical ends to obstruct all paths of leakage. Also, the residual microorganisms should be sealed inside the canal and prevented from accessing the periapical tissue. Application of sealer is imperative for a successful endodontic treatment. Sealer enters into the accessory canals and fills the fine gaps between the canal wall and root filling material.^1,2^

 The root end filling material and the root canal sealer should be able to bond to root dentin, and provide a hermetic seal. Optimal biocompatibility, solubility in a solvent and insolubility in tissue fluids, short setting time, not causing discoloration, bacteriostatic properties, and optimal radiopacity without polymerization shrinkage are among other requirements of endodontic sealers.^3^

 Biocompatibility is a requirement for dental material, as any toxic components present in them might induce irritation or degeneration of the surrounding tissues. Subcutaneous implantation in rats is among the most commonly employed methods to assess the local reactions caused by endodontic sealers.^4,5^ The toxic ingredients of sealers can irritate the periapical tissue or even damage it. Therefore, biocompatibility of endodontic sealers is a critical factor to consider.^6-8^

 To date, no ideal sealer has been introduced to the market. Despite the production of new sealers with mineral trioxide aggregate and silicon base and ceramic sealers with calcium phosphate base, resin sealers are still preferred in dental clinics.^9^ Among the commonly used sealers, AH26 is a commonly used epoxy resin sealer in endodontics. AH-Plus is the newer version of AH26 sealer with improved properties. AH26 sealer is a bis-phenol epoxy resin sealer that uses hexamethylenetetramine (methenamine) for polymerization. Low shrinkage is an advantage of AH26. However, it releases formaldehyde following polymerization, which is a major drawback of this sealer.^10^

 The more recent AH-Plus sealer has all the optimal properties of AH26 without its drawbacks such as formaldehyde release. Formaldehyde released from endodontic sealers can have adverse consequences. Paresthesia of the mandibular nerve has been attributed to formaldehyde release from endodontic sealer in overfilled root canals. Evidence exists regarding formaldehyde release after setting of AH26 sealer. However, formaldehyde release from AH-Plus is minimal.^11^

 Despite the introduction of new sealers with mineral trioxide aggregate base and silicon and ceramic sealers with calcium phosphate base, resin sealers are still highly popular in endodontics. Resil is a new experimental epoxy resin sealer that contains hexamethylenetetramine, bismuth oxide, titanium oxide, calcium tungstate and zirconium oxide. It had some superiority over AH26 sealer including shorter setting time and less cytotoxicity. It was highly similar to AH26 and AH-Plus. Resil had acceptable flow, solubility and film thickness according to ANSI/ADA No.57 and ISO 6867 (2012) standards.^9,12,13^ However, studies on its biocompatibility are lacking. Considering the common application of subcutaneous implantation of dental materials in rats for evaluation of their biocompatibility, this study sought to assess the biocompatibility of Resil experimental sealer in comparison with AH-Plus and AH26.

## Materials and Methods

 This experimental study used tissue specimens obtained from 12 rats between 4 to 6 weeks, weighing 400 to 500 g after implantation of polyethylene tubes containing Resil experimental sealer (institute for color science and technology, Tehran, Iran), AH-Plus (Dentsply, De Trey, Konstanz, Germany), AH26 (Dentsply, De Trey, Konstanz, Germany), AH-Plus, AH26, and an empty control tube to assess their biocompatibility.

 Sample size was calculated to be 12 rats (n = 6 for assessment at each time point) according to previous studies.^14-16^

 The inclusion criteria were male Wistar rats weighing 400 to 500 g between 4 to 6 weeks of age with no history of previous intervention.

 Eligible rats were kept in rooms with controlled temperature of 25°C, and 12-hour light/12-hour dark cycles with ad libitum access to food and water. The rats were kept under the same conditions for 1 week for the purpose of acclimation, and their weight and health status were monitored during this time period. A total of 48 polyethylene tubes were used in this study for implantation in 12 rats (each rat received 4 tubes). Of 48 tubes, 12 remained empty and served as the control group. Of the remaining 36, 12 were filled with AH-Plus (Dentsply Maillefer), 12 were filled with AH26 (Dentsply Maillefer) and 12 were filled with Resil experimental sealer. The sealers were mixed according to the manufacturers’ instructions. Resil experimental sealer is composed of a powder and a liquid component. The liquid component is made of epoxy resin with optimal viscosity. The powder is composed of calcium tungstate, bismuth oxide, titanium oxide, amine powder and zirconium oxide with adequate ratios.

 The rats were anesthetized by intramuscular injection of ketamine (Rotex Medica, Germany) and xylazine (Alfasan, Netherlands) with 3:1 ratio. After disinfection with 5% iodine solution, the skin of the back was shaved and disinfected again with 5% iodine. Next, local anesthesia was administered using 2% lidocaine HCl (Persocaine; Daroupakhsh, Iran). A 1-centimeter incision was made using a #15 scalpel. The skin was reflected to create a pocket at the right side of the incision. A polyethylene tube was implanted in the created space and the skin was sutured using 4-0 silk sutures. Four polyethylene tubes (containing the three sealers and one empty tube) were implanted subcutaneously in each rat. The rats received 400 mg cephalexin for 1 week and 100 mg acetaminophens for 2 days, postoperatively. The medications were dissolved in the drinking water of rats. Also, tetracycline ointment was applied over the incision site for 3 days, postoperatively. At 7 and 30 days after implantation of tubes, the rats were anesthetized by intramuscular injection of ketamine and xylazine, and sacrificed by overdose in a CO chamber. The polyethylene tubes along with the adjacent tissues were resected and fixed in 10% formaldehyde for 24 hours. Transverse sections were made at three zones along the surgical incision line namely at the initiation, at the middle and at the end of defect. After embedding the tissue specimens in paraffin blocks, each sample was sectioned into a minimum of nine 4-µ-thick slices at the three areas. The specimens were stained with hematoxylin and eosin, and histologically analyzed by a pathologist blinded to the group allocation of specimens. Tissue reactions at the open ends of the tubes were evaluated and categorized based on the number of inflammatory cells (neutrophils, macrophages, etc.) as follows.^17^

 Score 0: Absence of inflammatory cells or their small number with no inflammatory reaction

 Score 1: Presence of less than 25 inflammatory cells and mild reactions

 Score 2: Presence of 25-125 inflammatory cells and moderate reactions

 Score 3: Presence of ≥125 inflammatory cells and severe reactions

 The percentage and severity of inflammation was categorized as follows:

 Score 0: Less than 10% (very low)

 Score 1: Between 10% and 30% (low)

 Score 2: Between 30% and 50% (moderate)

 Score 3: Over 50% (high)

 Fibrotic capsules with < 150 µ thickness were considered thin and those with > 150 µ thickness were considered as thick.^16^ Presence/absence of necrosis, granulation tissue, giant cells, and bleeding was also reported (dichotomized as presence/absence). The mean number of cells in each experimental group was counted in 10 separate areas under a microscope (Nikon eclipse 50i, made in Germany) at ×400 magnification and reported.^17^

 Data were analyzed using SPSS version 24 (SPSS Inc., IL, USA). The differences between the variables in the four groups at 7 and 30 days were analyzed using the chi-square test. Within-group comparisons (between 7 and 30 days) were also performed by the chi-square test. Level of significance was set at 0.05.

## Results

Table 1 presents the measured parameters at 7 and 30 days after implantation of tubes in the four groups. At 7 days after implantation of tubes, no significant difference was noted in the capsule thickness, percentage and severity of inflammation, number of lymphocytes, macrophage, eosinophil and mast cells, presence or absence of giant cells, bleeding, necrosis and granulation tissue between the Resil and control, AH26 or AH-Plus groups (*P* > 0.05). however, the neutrophil count in AH-Plus group was significantly higher than Resil group (*P* = 0.02) while difference between Resil and control (*P* = 0.63) and AH26 sealer (*P* = 0.93) was not significant.

**Table 1:** Measured parameters at 7 days and 30 days after implantation of tubes in the four groups

**Variables/categories** **No. (%)**		**Control**		**Resil**		**AH26**		**AH-Plus**	
		**7 days**	**30 days**	**7 days**	**30 days**	**7 days**	**30 days**	**7 days**	**30 days**
Capsule thickness	Thin	6 (100)	2 (33.3)	6 (100)	1 (16.7)	6 (100)	3 (40)	5 (83.3)	3 (50)
	Thick	0 (0)	4 (66.7)	0 (0)	5 (83.3)	0 (0)	3 (60)	1 (16.7)	3 (50)
Percentage of inflammation	< 10	2 (33.3)	5 (83.3)	2 (33.3)	5 (83.3)	1 (16.7)	5 (83.3)	1 (16.7)	5 (83.3)
	10-30	3 (50)	1 (16.7)	0 (0)	0 (0)	1 (16.7)	0 (0)	3 (50)	1 (16.7)
	30-50	1 (16.7)	0 (0)	4 (66.7)	1 (16.7)	4 (66.7)	1 (16.7)	2 (33.3)	0 (0)
	> 50	0 (0)	0 (0)	0 (0)	0 (0)	0 (0)	0 (0)	0 (0)	0 (0)
Severity of inflammation	Very low	2 (33.3)	5 (83.3)	2 (33.3)	5 (83.3)	1 (16.7)	5 (83.3)	1 (16.7)	5 (83.3)
	Low	3 (50)	1 (16.7)	0 (0)	0 (0)	2 (33.3)	0 (0)	3 (50)	1 (16.7)
	Moderate	1 (16.7)	0 (0)	4 (66.7)	1 (16.7)	3 (50)	1 (16.7)	2 (33.3)	0 (0)
	High	0 (0)	0 (0)	0 (0)	0 (0)	0 (0)	0 (0)	0 (0)	0 (0)
Number of neutrophils	Almost zero	1 (16.7)	6 (100)	1 (16.7)	6 (100)	1 (16.7)	6 (100)	4 (66.7)	6 (100)
	< 25	5 (83.3)	0 (0)	3 (50)	0 (0)	4 (66.7)	0 (0)	2 (33.3)	0 (0)
	25-125	0 (0)	0 (0)	1 (16.7)	0 (0)	1 (16.7)	0 (0)	0 (0)	0 (0)
	> 125	0 (0)	0 (0)	1 (16.7)	0 (0)	0 (0)	0 (0)	0 (0)	0 (0)
Number of lymphocytes	Almost zero	0 (0)	0 (0)	0 (0)	0 (0)	0 (0)	0 (0)	0 (0)	1 (16.7)
	< 25	6 (100)	6 (100)	6 (100)	6 (100)	6 (100)	6 (100)	6 (100)	5 (83.3)
	25-125	0 (0)	0 (0)	0 (0)	0 (0)	0 (0)	0 (0)	0 (0)	0 (0)
	> 125	0 (0)	0 (0)	0 (0)	0 (0)	0 (0)	0 (0)	0 (0)	0 (0)
Number of eosinophils	Almost zero	3 (50)	6 (100)	4 (66.7)	6 (100)	5 (83.3)	6 (100)	4 (66.7)	6 (100)
	< 25	3 (50)	0 (0)	2 (33.3)	0 (0)	1 (16.7)	0 (0)	2 (33.3)	0 (0)
	25-125	0 (0)	0 (0)	0 (0)	0 (0)	0 (0)	0 (0)	0 (0)	0 (0)
	> 125	0 (0)	0 (0)	0 (0)	0 (0)	0 (0)	0 (0)	0 (0)	0 (0)
Number of macrophages	Almost zero	0 (0)	3 (50)	0 (0)	1 (16.7)	0 (0)	1 (16.7)	0 (0)	3 (50)
	< 25	6 (100)	3 (50)	6 (100)	5 (83.3)	6 (100)	5 (83.3)	6 (100)	3 (50)
	25-125	0 (0)	0 (0)	0 (0)	0 (0)	0 (0)	0 (0)	0 (0)	0 (0)
	> 125	0 (0)	0 (0)	0 (0)	0 (0)	0 (0)	0 (0)	0 (0)	0 (0)
Number of giant cells	Almost zero	3 (50)	4 (66.7)	3 (50)	1 (16.7)	3 (50)	0 (0)	3 (50)	3 (50)
	< 25	3 (50)	2 (33.3)	3 (50)	4 (66.7)	3 (50)	3 (50)	3 (50)	1 (16.7)
	25-125	0 (0)	0 (0)	0 (0)	1 (16.7)	0 (0)	3 (50)	0 (0)	2 (33.3)
	> 125	0 (0)	0 (0)	0 (0)	0 (0)	0 (0)	0 (0)	0 (0)	0 (0)
Bleeding	Absence	3 (50)	4 (66.7)	1 (16.7)	3 (50)	1 (16.7)	3 (50)	1 (16.7)	4 (66.7)
	Presence	3 (50)	2 (33.3)	5 (83.3)	3 (50)	5 (83.3)	3 (50)	5 (83.3)	2 (33.3)
Necrosis	Absence	3 (50)	5 (83.3)	2 (33.3)	5 (83.3)	1 (16.7)	6 (100)	4 (66.7)	6 (100)
	Presence	3 (50)	1 (16.7)	4 (66.7)	1 (16.7)	5 (83.3)	0 (0)	2 (33.3)	0 (0)
Granulation tissue	Absence	0 (0)	4 (66.7)	0 (0)	4 (66.7)	0 (0)	2 (33.3)	0 (0)	4 (66.7)
	Presence	6 (100)	2 (33.3)	6 (100)	2 (33.3)	6 (100)	4 (66.7)	6 (100)	2 (33.3)
Number of mast cells	Almost zero	5 (83.3)	6 (100)	5 (83.3)	5 (83.3)	5 (83.3)	5 (83.3)	5 (83.3)	6 (100)
	< 25	1 (16.7)	0 (0)	1 (16.7)	1 (16.7)	0 (0)	1 (16.7)	1 (16.7)	0 (0)
	25-125	0 (0)	0 (0)	0 (0)	0 (0)	1 (16.7)	0 (0)	0 (0)	0 (0)
	> 125	0 (0)	0 (0)	0 (0)	0 (0)	0 (0)	0 (0)	0 (0)	0 (0)

**Figure 1:**
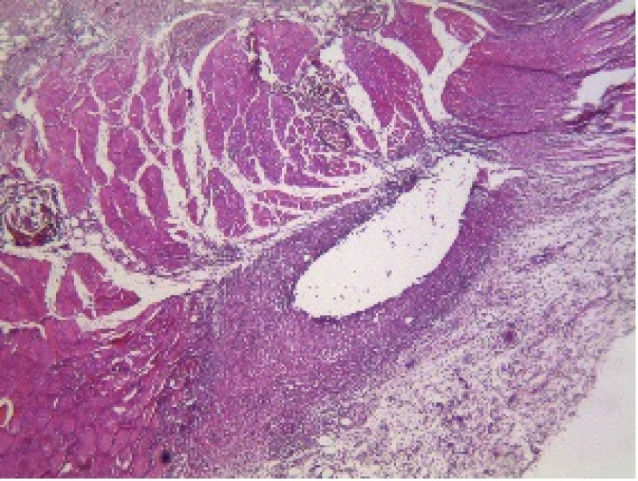
Histopathological view of granulation tissue at ×40 magnification (AH-Plus).

**Figure 2:**
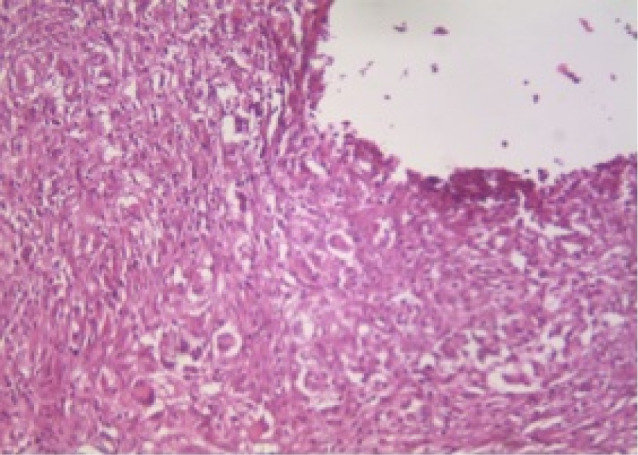
Histopathological view of giant cells at ×200 magnification (Resil).

 At 30 days after implantation of tubes in the four groups, all of the measured parameters did not show significant difference (*P* > 0.05). Figures 1 and 2 indicate the histopathological view of granulation tissue at ×40 magnification and giant cells at ×200 magnification.

**Figure 3:**
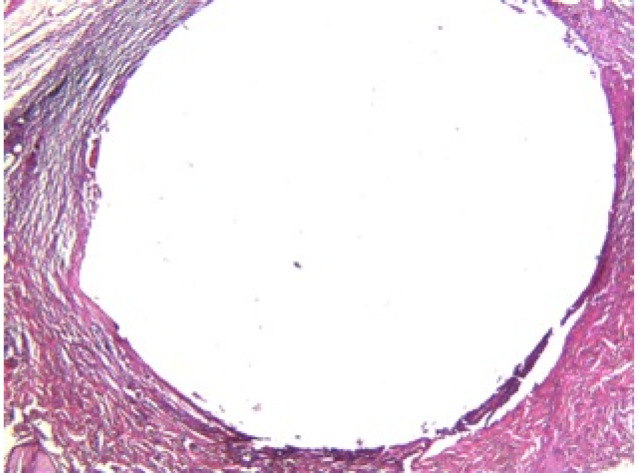
Histopathological view of inflamed granulation tissue at ×40 magnification (AH26).

Table 2 presents the statistical differences at 7 and 30 days after implantation of tubes in the four groups. The granulation tissue in the control group at 30 days was significantly lower than that at 7 days (*P* = 0.05). In Resil group, tissue fibrosis (*P* = 0.01), number of neutrophils (*P* = 0.00) and granulation tissue (*P* = 0.05) at 30 days were significantly lower than the corresponding values at 7 days. AH-26 group showed significantly lower number of neutrophils (*P* = 0.02) and tissue necrosis (*P* = 0.02) at 30 days compared with 7 days (Figure 3). In AH-Plus group, the granulation tissue at 30 days was significantly lower than that at 7 days (*P* = 0.05).

**Table 2:** Statistical differences at 7 and 30 days after implantation of tubes in the four groups

**Variable**	**Group**			
	**Control**	**Resil**	**AH26**	**AH-Plus**
Fibrosis	0.07	0.01	0.19	0.79
Percentage of inflammation	0.78	0.32	0.16	0.32
Severity of inflammation	0.78	0.32	0.16	0.32
Number of neutrophils	0.08	0.00	0.02	0.93
Number of lymphocytes	1.00	1.00	1.00	0.49
Number of eosinophils	0.24	0.72	0.99	0.72
Number of macrophages	0.21	0.98	0.98	0.21
Number of giant cells	1.00	0.85	0.13	0.98
Bleeding	0.99	0.93	0.93	0.64
Necrosis	0.84	0.41	0.02	0.84
Granulation tissue	0.05	0.05	0.75	0.05
Number of mast cells	0.99	1.00	0.99	0.99

## Discussion

 This study sought to assess the biocompatibility of Resil, an experimental epoxy resin sealer, in comparison with AH26 and AH-Plus in rats. Selection of AH26 and AH-Plus sealers for the purpose of comparison in this study was due to the fact that they are among the most commonly used resin-based endodontic sealers. AH-Plus has been used as the gold standard sealer as well.^10^

 Resil experimental endodontic sealer that was used in this study has zirconium and tungsten in its composition to confer radiopacity. The porosity of this sealer is due to the presence of zirconium oxide particles in its composition. Thus, sealer particles are easily detectable in the resin matrix.^13,18^ The cytotoxicity, setting time, flow rate, film thickness, radiopacity and solubility of this experimental sealer have been previously studied, and the results have shown that Resil has shorter setting time and lower cytotoxicity than AH26. Structurally, Resil highly resembles AH26 and AH-Plus. Also, Resil had acceptable flow, solubility and film thickness according to ANSI/ADA No.57 and ISO 6876 (2012) standards. Its radiopacity is higher than 3 millimeter of aluminum according to ANSI/ADA No.57 and ISO 6876 (2012) standards.^13^Another study compared the cytotoxicity of Resil and AH26 in vitro and reported that the cytotoxicity of both sealers decreased over time. Maximum cytotoxicity belonged to 100% concentration of Resil, and its cytotoxicity in 1% and 10% concentrations was low.^12^

 Despite the significant cytotoxic effects of AH26, it is routinely used in the clinical setting. However, it should be noted that a cytotoxic material in vitro may not be cytotoxic in vivo. Thus, in vivo studies are required for assessment of cytotoxicity of endodontic sealers and their success rate. Biocompatibility of sealers is an important factor to consider prior to their use in the clinical setting.^6-8^ The current results showed that the biocompatibility of Resil sealer was comparable to that of AH-Plus and AH26 such that Resil group had no significant difference with AH-Plus and AH26 in severity and percentage of inflammation, the number of neutrophils, eosinophils, macrophages, mast cells and giant cells, bleeding, and granulation tissue formation. At 30 days, the percentage and severity of inflammation, the number of neutrophils, eosinophils and giant cells, and the granulation tissue in Resil group were comparable to AH-Plus. Also, Resil was similar to AH26 regarding the percentage and severity of inflammation, the number of neutrophils, eosinophils, lymphocytes, macrophages and mast cells, as well as bleeding. Ashraf et al^13^evaluated the physical and chemical properties and biocompatibility of two experimental endodontic sealers in comparison with AH26 and reported that the experimental sealers had properties in accord with the ISO standards. Angelo Cintra et al^19^ evaluated the cytotoxicity and biocompatibility of an experimental endodontic epoxy resin sealer containing calcium hydroxide (Sealer Plus) and compared it with AH-Plus, Endofill and Simpliseal. They adopted a methodology similar to ours and reported thin fibrotic capsule around the tubes at 7 and 30 days in all sealer groups except for Simpliseal. They reported that Sealer Plus experimental sealer had higher biocompatibility and cell viability than other experimental sealers. Simsek et al^20^ assessed the biocompatibility of an experimental epoxy resin endodontic sealer (Obtuseal) compared with AH-Plus with a methodology similar to ours. They reported a significant increase in infiltration of lymphocytes in both groups after 7 days. However, infiltration of macrophages was significantly higher in AH-Plus group. Infiltration of macrophages and lymphocytes decreased after 14 and 45 days. At the end of the study period, no significant difference was noted between AH-Plus and Obtuseal. They concluded that Obtuseal had an acceptable biocompatibility comparable to that of AH-Plus at 45 days. Similar to previous studies mentioned above, we compared Resil experimental sealer with AH-Plus and AH26 and found higher cytotoxicity of sealers at 7 days, which decreased at 30 days. Similarity in biocompatibility of Resil, AH-Plus and AH26 in our study may be due to the similarity of their particles. Another study compared the biocompatibility of a new ceramic-based sealer (GuttaFlow bioseal) with GuttaFlow 2 and AH-Plus. They implanted the sealer tubes subcutaneously in rats and sacrificed the rats at 8 and 30 days. They reported a reduction in inflammatory reactions at 30 days compared with 8 days and showed that all tested sealers had acceptable biocompatibility at 30 days.^21^ Our study also showed a reduction in severity and percentage of inflammation during the study period for all three sealers; however, these changes did not reach statistical significance. However, in Resil group, the neutrophil count, which is an index of inflammation around the site of implantation of tubes, significantly decreased at 30 days compared with 7 days. At 7 days, 66.7% of the rats in Resil and AH26 groups showed moderate level of inflammation while the level of inflammation was lower in AH-Plus group (50% of rats showed low and 33% showed moderate inflammation in this group). At 30 days, the level of inflammation decreased in all four groups such that 83.3% of rats in all groups showed very low level of inflammation. This finding indicates that the percentage of inflammation in Resil group was comparable to that in AH26 group and higher than that in AH-Plus group at 7 days; while at 30 days, it was comparable to AH26 and AH-Plus, which indicates reduction of cytotoxicity at 30 days after setting of sealers.

 This study had some limitations. It was an animal study and the results cannot be completely generalized to the clinical setting. Future randomized clinical trials are required to assess the properties of this new sealer in the clinical setting. Also, specific staining was not used in histopathological analysis of tissue specimens, which was another limitation of this study. Other properties of this sealer such as its bioactivity and antimicrobial properties should be evaluated in further investigations.

## Conclusion

 Resil experimental sealer showed appropriate biocompatibility at 7 and 30 days after subcutaneous implantation in rats comparable to AH26 and AH-Plus. Further clinical studies are required to confirm these results.

## Acknowledgments

 There is no financial support in this study. The authors deny any conflict of interest related to this study.

## Authors’ contribution

 AH and HS contributed to conception and design. SP contributed to all experimental work, data and statistical analysis, and interpretation of data. SHH performed surgical procedure in this study. AL conducted animal experiments. MAF involved in the preparation and evaluation of histopathological specimens. HS and ZS were responsible for overall supervision. SHP and ZA drafted the manuscript. All authors read and approved the final manuscript.

## Funding

 There are no sources of funding for research reported.

## Ethics Approval

 The study was conducted in accordance with the guidelines for the care and use of laboratory animals, and was approved by the ethics committee of Shahid Beheshti University of Medical Sciences (IR.SBMU.DRC.REC.1397.024).

## Competing Interests

 None declared.
